# The taming of sociodigital anticipations: AI in the digital welfare state

**DOI:** 10.3389/fsoc.2025.1556675

**Published:** 2025-05-14

**Authors:** Thomas Zenkl

**Affiliations:** Department of Sociology, University of Graz, Graz, Austria

**Keywords:** AI, algorithms, anticipation, public employment services, digital welfare state

## Abstract

Advanced algorithmic technologies and artificial intelligence (AI) are projected to profoundly impact public employment services (PES) and the delivery of labor market policies. Focusing on the perspectives of PES counselors in Austria, this study examines workers’ anticipations of future AI technologies. Based on face-to-face interviews (*n* = 23), it identifies and explores the concept of “taming” as a primary tactic for managing and appropriating uncertain AI futures within the limits of current institutional and bureaucratic structures. “Tamed” anticipations of advanced algorithms are rooted within challenging working conditions (insufficient resources and time for clients), reconfigurations of roles and agencies (administration of systems instead of supporting clients) and nested within transformations of techno-bureaucratic regimes (from street- over screen- to system-level bureaucracies), which they envision to rectify and repair. Taming is thus applied as a lens to observe practices of anticipation that navigate and contest precarities of daily work life beyond dichotomies of compliance and resistance. Despite the differences in tamed AI futures identified, the analysis shows corresponding properties: Tamed anticipations consolidate dependencies between human and machinic actors, demarcate what is (un-)tamable, and are inherently dialectical. Synthesizing structural problems and an imperative for human elements into futures that rely on advanced technologies, a truly “human” counseling situation is thought to be obtained through “machinic” means. To “tame” futures with and by anticipating certain AI technologies here means affirming ideas of a supposedly better working conditions (more humanity and margin of discretion) and regaining (or even improving) them through the computational means that are considered responsible for their loss.

## 1 Introduction

Building on historical evolutions of welfare regimes and developments of automated technologies, the digital welfare state constitutes a critical field for future applications of so-called artificial intelligence (AI, Dencik, 2022; Larasati et al., 2022; van Toorn et al., 2024). Pushed by the combined effects of the global COVID-19 pandemic and pursuing trends of digitalization, a spreading use of applications facilitating predictive algorithms increasingly transforms political economies of the welfare state (Collington, 2022) and affect the delivery of policies. In this context, use cases for the ongoing automation of public employment services (PES; Barnes et al., 2015; Desiere et al., 2019; Körtner and Bonoli, 2023) include interfacing clients, statistical profiling, data processing and analysis, as well as the matching, targeting, classification and segmentation of jobseekers (Desiere and Struyven, 2021; Etscheid et al., 2022). Despite their predominantly still speculative effects, it is expected that “advanced technologies will be widespread in the next decade, pushing public employment services into the next wave of government services digitalization” (International Labour Organization, 2022, p. xi).

Anticipations of future technologies are critical for understanding expected usages as well as present negotiations and embedding: futures are routine elements of thought and an integral part of how societies shape their practice (Appadurai, 2013, p. 292). They are no neutral, sterile or technical spaces, but “shot through with affect and with sensation” (Appadurai, 2013, p. 287) that give gravity, traction, and texture to certain developments, while neglecting others. Speculation on possible futures and their anticipation links the future to the present and “creates material trajectories of life that unfold as anticipated” (Adams et al., 2009, p. 248). As “the palpable effect of the speculative future on the present” (Adams et al., 2009, p. 247), anticipation “pervades the ways we think about, feel and address our contemporary problems.” (Adams et al., 2009, p. 248).

Despite a growing body of literature outlining applications for AI and their possible future effects within continuously digitizing data-driven welfare states (Meijer et al., 2021; Braunsmann et al., 2022; Busemeyer, 2022; Dencik, 2022; Carney, 2023; Kaun et al., 2023) or PES (Allhutter et al., 2020; Zejnilović et al., 2020; Desiere and Struyven, 2021; Kern et al., 2021; Petersen et al., 2021), little attention has been paid to how counselors anticipate the impacts of AI transforming their workplaces. As “the routines they establish, and the devices they invent to cope with uncertainties and work pressures, effectively become the public policies they carry out” (Lipsky, 1980, p. xiii), these workers are not merely rule-abiding analog interfaces of technologized bureaucracies, but partake in influencing, repairing and maintaining them (Kaun and Liminga, 2023). Thus, workers’ practices (of anticipation) are crucial elements both for enacting current policies and shaping the ones to come.

While anticipating certain futures holds transformational potential, it is confined within the limits of powerful discourses, shaped by the institutional logics surrounding it (Markham, 2021) and thus never entirely contingent. In the case of PES, the speculations guiding possible future applications of AI are informed by the threat of job redundancy (Ouchchy et al., 2020; Köstler and Ossewaarde, 2022), fueled by workers’ fears of marginalization resulting from increasingly accurate algorithmic predictions (Körtner and Bonoli, 2023) or expected to reinforce various existing forms of inequality and discriminations (Allhutter et al., 2020; Desiere and Struyven, 2021; Kern et al., 2021; Busemeyer, 2022). Contradicting claims on technological impacts include employees’ empowerment through automation (Giesbrecht et al., 2017; Dolata et al., 2020) or bear promises of improved public services by increasing fairness, efficiency, and efficacy. Already today, advances in information and communications technologies (ICTs) in PES are shaping governance regimes: undermining the former discretion of “street-level bureaucrats” (Lipsky, 1980; Zacka, 2017), technological logics penetrating formerly human domains are regarded to induce organizational shifts towards what has been called “screen-” or “system-level bureaucracies” (Bovens and Zouridis, 2002).

In the context of these conflicting technological expectations, attributions, negotiations, institutional constraints and bureaucratic transformations outlined, workers’ anticipation must be understood as a mode of appropriating unknowable and potentially precarious futures, or what Adam and Groves (2007, p. 6) describe as “taming”: knowledge practices “providing structural security for the daily and seasonal rounds of social life.” Such “taming” of sociodigital futures might be “an action aiming to gain control over a situation perceived as threatening in an uncontrolled state,” “an attempt to adapt something uncontrolled to one’s own needs,” but also as “a slowing down of digitalization, carried out by skeptical actors who perceive digitalization as uncontrolled and threatening” (Erichsen, 2021, p. 1).

Building on the argument that technologies “must be tamed to serve the public good” (Editorial Team, 2021, p. 1), following discussion how algorithmic technologies can (Röhl, 2021; Schmidt, 2021) or cannot be tamed (Gulson et al., 2021), “taming” is facilitated here as an analytical lens to ask how workers anticipate potentially disruptive future technologies, what institutional conditions and logics of bureaucratic regimes such practices of anticipation frame and what properties, e.g., regarding professional roles and agencies, such taming includes. Examining how workers project themselves into uncertain futures and analyzing their anticipations as practices of “taming” thus seeks to interrogate how, through the lens of AI futures, contemporary problems are addressed, perceived, and solved; It reveals how “taming” is employed as empowerment over and contestation of dominant narratives surrounding the expected consequences of AI, appropriating uncertainties and relegating machinic agencies of sociotechnical algorithmic systems prior to their implementation.

As an “act too often neglected,” the fox in Saint-Exupéry’s (1971, p. 80) famous “The little prince” notes that taming “means to establish ties.” It is these anticipated ties to AI technologies within workers’ anticipations that are central to this study. Such taming, as will be shown, forges dependencies (rather than rejects novel technologies), demarcates what is tamable (and what is not) and, by synthesizing futures that appear as a reciprocal rapprochement and as mutual reconciliation, is inherently dialectic.

First, the analysis reflects upon the concepts outlined and discusses the conditions of progressive automation of the digital welfare state and datafied practices in PES before deriving the research questions. Methodological considerations are followed by describing workers’ perceptions of contemporary ICTs in Austria’s PES agency and their effects regarding the reconfigurations of professional roles and practices. Based on experiences concerning an ongoing proliferation of digital systems and resulting notions of dehumanization and restriction of discretionary margins, a subsequent section discusses how anticipations of AI futures overcome these tensions by “taming” certain aspects of technological developments. A final section identifies the properties of such taming: the creation of dependencies, the demarcation of the (un-)tamable, and its inherent dialectical character.

## 2 Related work

This section reflects on “taming” as means of coping with uncertain futures, highlights that neither the areas of application nor anticipations of future AI technologies unfold in a social vacuum, examines past and ongoing transformations of governance regimes within PES and derives research questions.

### 2.1 Taming uncertain futures

Futures are both tense and ambiguous. As “human preoccupations that shape the future as a cultural fact, as a form of difference” (Appadurai, 2013, p. 286), people discursively engage in everyday processes of world- and future-making by anticipating certain trajectories. Such “concrete imaginaries of the future to come mold and shape who we are, and the directions in which we wish, can and will go” (Cantó-Milà and Seebach, 2015, p. 198). Understanding the making of futures means asking about the place where probable and possible, desirable and undesirable moments converge. As the tensions arising from this conjunction configure specific cultural horizons, their examination allows for analyzing underlying assumptions and to interrogate them with regard to their containing strategies of refusal (Adams et al., 2009, p. 60), their “escape routes” (Papadopoulos et al., 2015) through which they remake the present and sabotage the future (Cooper, 2006).

However, facing uncertainties of technological and bureaucratic transformations (as in the case of PES) must not culminate in outright insurgency. Taming, as a mode of appropriating unknowable and precarious futures, is a specific way of practically anticipating between ethics of possibility (how the future could and should be) and probability (how it is most likely going to be, Appadurai, 2013, p. 295). Ordering “the insecurities of the realm beyond experience” (Adam and Groves, 2007, p. 25), taming makes daily life (and anticipated future work scenarios) less precarious. Just like “aspiring,” taming functions “as a navigational tool, through which people can chart their way out of a position of entrapment” (Markham, 2021, p. 19), however, without resorting to seemingly utopian subversion. Taming both repairs and reaffirms the tensions and irritations it arises from.

While discourses of technological developments in the public sector (and beyond) are often framed as questions of “humans against machines” (Dressel and Farid, 2018; Lin et al., 2020; Körtner and Bonoli, 2023), the perspective of taming renounces human-machine binaries by highlighting reciprocal future adaptations and approximations. As “dominant and oppositional forces are simultaneously present when people make sense of algorithmic relations” (Ruckenstein, 2023, p. 66), questioning futures towards peoples’ acts of taming seeks not to negate tensions, but to investigate how tensions are being dealt with.

### 2.2 The techno-bureaucratic transformations of PES

Beyond its function of providing social services, the welfare state here is understood as specific mode of government that manifests as distinctive rationalities, functions, effects, and practices (Garland, 2014). Arising in the late 19th and early 20th century, welfare states are not only “fundamental forces in the organization and stratification of modern economies” (Kolberg and Esping-Andersen, 1991, p. 25), but have “profound influence on such social institutions as the labor market, the family, the class structure, the systems of distribution and redistribution, the normative structure, and gender relations” (Kolberg and Esping-Andersen, 1991, p. 5) in capitalist societies. Today, the “digital” welfare state refers to a particular state formation that operates by facilitating a broad spectrum of data-driven technologies, such as AI, algorithmic or decision-support systems (van Toorn et al., 2024). With the advances of ICTs into welfare practices since the 1960ies, sophisticated analyses facilitating “big and smart data” have today become central to all levels of welfare governance, further propelling digital transformations (Pieterson, 2019; Weishaupt, 2023). Reflecting neoliberal disruptions of “New Public Management” (NPM, Kamp, 2016), data intense technologies enabled and pushed bureaucratic governance driven by input/output optimization rather than on procedural compliance with public law, introduced novel measures (customer satisfaction and quality management) and technologically supported forms of service delivery (Weishaupt, 2023, p. 361). Importantly, these developments must be perceived as changes *within* continuity rather than as a radical disruption and renunciation of prior regimes (Fussey and Roth, 2020). Welfare states are “intricately tied to the evolution of statistical forms of knowledge, methods and technologies aimed at delineating and managing populations, particularly the poor” (van Toorn et al., 2024, p. 511). Their digital adaptions thus build upon and proceed with a historical lineage of social policy and the collection of information on citizens (Higgs, 2004).

Labor market policy – and its delivery by PES – is one of the crucial arenas for mediating the relationship between democracy and capitalism in the political economy of those affluent countries with an established welfare state (Clegg and Durazzi, 2023). In the wake of welfare institutions shifting to consumer-oriented service providers (Penz et al., 2017), many of the formerly governmental agencies were remodeled into semi-autonomous organizations in the mid-1990s (Ludwig-Mayerhofer and Wroblewski, 2004). In the case of public employment, these PES agencies are tasked with integrating jobseekers into the labor market and usually provide them with various services (e.g., benefits, insurance, counseling).

Notwithstanding agencies’ statutory scope of duties, Lipsky’s (1980) notion of “street-level bureaucracies” suggests to understand public policy not exclusively through the decision-making arenas of high-ranking administrators or legislation, but rather through the practices of those “street-level bureaucrats” that constitute the interface between the state and the public (Zacka, 2017). Contrary to a Weberian (1978/1921) ideal type of formalized processes within calculable bureaucracies, this approach centers the agency that the state’s frontline workers – teachers, police officers, and caseworkers – exercise, highlighting their margins of discretion in delivering policies. Focusing on the moment in which “the state meets the street” (Zacka, 2017) emphasizes the many ways in which policy is produced situationally, shaped and mediated by frontline workers.

Ongoing processes of digitalizing the welfare services also affect the delivery of labor market policies. As contacts between frontline workers and citizens increasingly become mediated via (or occur in presence with) various technologies, they foster shifts towards “screen-level bureaucracies” (Bovens and Zouridis, 2002). Public servants no longer “take the streets,” but become connected to their organization via electronic forms and fixed templates and have to provide for growing needs for data (Rapson, 2018; Weishaupt, 2023). Data warehouse applications were introduced to meet political requirements of performance measuring and demands of internal controlling.

Further intensifications hint at shifts towards “system-level bureaucracies,” in which the members of organizations are primarily concerned with mediating between systems rather than interfacing clients (Bovens and Zouridis, 2002, p. 10). Irrespective of this analysis’ accuracy, the ongoing quantification of worker-client relationships along measurable indicators continues to “squeeze” the discretionary margin in rules and regulations that the role of “street-level bureaucrats” once encompassed. As gaps between policy and practice that formerly needed to be “bridged” by workers increasingly become supported by statistical evaluations and computationally compared to the decisions made in other cases, they affect professional roles, agencies, expectations, practices, and ultimately the discretion and the affective subjectivation of those delivering policies at the frontlines (Caswell et al., 2010; Kamp, 2016; Penz et al., 2017).

### 2.3 Datafied PES

Frontline workers’ tasks in PES are usually classified along the stages of a counseling situation, namely profiling (classification of jobseekers), targeting (selection of labor market programs) and matching (finding suitable job opportunities), all of which are increasingly supported and shaped by ICT (Broecke, 2023; van Berkel, 2023) and follow a growing “data imperative” (Schildt, 2020) in the public sector. Recent research on the algorithmic enhancement of PES has focused on the discursive forming of algorithms (Braunsmann et al., 2022), how algorithms and AI are made “actionable” (Büchner and Dosdall, 2021), and how frontline workers relate to automated technologies (Arni and Schiprowski, 2015; Grundy, 2015; Körtner and Bonoli, 2023). These investigations attribute to caseworkers a general unease to use algorithmic applications (Barnes et al., 2015; Grundy, 2015), highlight their discomfort with their professional role being transformed from a human service/social work function to a more administrative/policing role (McDonald and Marston, 2006), and have revealed resistances that arise from the implementation of specific algorithmic technologies (Arni and Schiprowski, 2015; Barnes et al., 2015). Depending on the institutional setting and scenarios of their usage, they are either perceived as to assist frontline workers in making decisions and support clients in gaining access to services, or as complicit in asserting an agency’s agenda (Ammitzbøll Flügge et al., 2021), producing data-driven accounts that become increasingly difficult to contest (Holten Møller et al., 2019).

As “street-level algorithms” (Alkhatib and Bernstein, 2019) enter the counseling situation, technologies assume tasks and decisions historically entrusted to street-level bureaucrats. Algorithmic applications increasingly substituting margins of decisions (Pieterson, 2019) represents a continuity in agencies’ efforts to make “street-level bureaucrats more accountable by reducing their discretion and constraining their alternatives” (Lipsky, 1980, p. 162). Obtaining accountability from counselors by technologically mediating and intensifying management control, these developments manifest as colonization of computational logics and reflect intimate and growing entanglements of bureaucratic and algorithmic regimes (Jarke et al., 2024).

While algorithmic assessments reinforce the trend of demanding accountability through automated procedures, their practical application and the devaluating of professional knowledges was found leading to “foot-dragging, gaming, and open critique” (Christin, 2017, p. 11) as attempts to preserve margins of discretion. Frontline workers negotiate both “curtailing” and “empowering” aspects of ICT in their workplace (Hansen et al., 2018) and find ways of “coping” (Dolata et al., 2020). Despite manifold techno-bureaucratic transformations, workers’ practices of maintenance, repair, and care, continue to affect the delivery of public services. Bernhard and Wihlborg (2022) emphasize public service employees’ crucial role in mediating between digital services and clients, in bridging “digital divides” and promoting inclusion. Yet, such mediation is often accompanied by experiencing conflicts between the various expectations and assumptions of stakeholders and systems (Boulus-Rødje, 2019), affordances of technological infrastructures, and the policies that caseworkers have to balance (Dolata et al., 2020).

### 2.4 Research questions

With algorithmic frictions arising in PES, “people have to find ways to tolerate the tension and live with them” (Ruckenstein, 2023, p. 66). In conjunction with narratives in which possible empowerments are discursively overshadowed by the perceived negative consequences of AI’s expansion, the question arises as to how those affected by potential disruptions manage the uncertainties that accompany these transformations. Between top-down narratives of future technologies and personal experiences that “distort the way algorithms are experienced and lived with” (Ruckenstein, 2023, p. 67), it is frontline workers’ anticipations of certain developments that inform present and guide trajectories of future practices. Thus, the questions deriving from the proposed perspective are: How do frontline workers in PES appropriate and tame AI futures? What are the properties of such taming, what projections of contemporary perceptions are they rooted in and what elements of anticipated futures does taming address?

Considering anticipations through the lens of taming allows to ask how, why, and in what ways workers within uncertain conditions appropriate futures and “project themselves into the realm of the not yet” (Adam and Groves, 2007). While some see the taming of beliefs around seemingly “wild” technologies necessary in order to acquire agency in digital literacy education (Schmidt, 2021) and highlight the importance of taming for re-imagining and regulating AI (Röhl, 2021), the perspective offered here seeks to analyze workers’ efforts of taming as everyday knowledge practice providing structural security in the face of uncertainty (Adam and Groves, 2007, p. 6).

## 3 Method

Twenty-three semi-structured face-to-face interviews with employees of the Austrian PES agency (Arbeitsmarktservice, AMS) were conducted between August and October 2023 as an exemplary case of a European digital welfare state formation. While Austria’s “conservative” (Esping-Andersen, 1990) or “continental” (Bonoli, 1997) welfare regime is characterized by displacing the market as a provider of welfare while preserving status differentials, turns to NPM during the mid-1990s led to the establishment of post-bureaucratic service agencies (Penz et al., 2017). Founded as a semi-autonomous public service company in 1994, the AMS reflects the rise of new managerialism approaches in the field of unemployment and accompanying realignments toward customer-oriented activation regimes (Ludwig-Mayerhofer and Wroblewski, 2004).

Interviews were held in person at nine regional branches of three Austrian federal states which were chosen to represent the variety of urban and rural regions. After selection, branch managers were contacted, informed about the project, encouraged to nominate interviewees from among their employees and to take into account the inclusion of different perspectives (gender identity, years of working experience, position). Despite efforts to diversify, this sampling strategy is biased towards those employees interested in participating, likely due to prior knowledge or a certain opinion regarding the subject of the study. Further biases may result from managers’ pre-selection in terms of nominees’ expected attitudes and skills towards new technologies. Interviews were conducted in German language, at participants’ offices, and within their working hours.

The sample consisted of 17 front-line workers within different areas of expertise (job- and career information, counseling of job seekers with or without special needs, service for companies) and additional 6 participants in executive positions (heads or deputy heads of branches and/or departments), which all had previously worked in positions that involved dealing directly with clients. It represented a broad spectrum of work experiences (from trainees to employees of more than 30 years) and a balanced gender ratio (12 female, 11 male), with interviews averaging at 51 min. Focusing mainly on technological transformations within the counseling situation, the term “counselor” used in the remainder of the paper encompasses frontline- and caseworkers and their “counseling” experiences made at the front-line of delivering services.

The interview guide for the semi-structured interviews comprised three thematic blocks to be covered throughout the interview: (1) current perceptions and experiences with the use of technologies in the working environment, (2) expectations, hopes and fears associated with future technologically induced transformations of the workplace, and (3) assessments of specific use cases implementing AI. Each thematic block included a list of possible conversation starters and sample questions that were used to bridge or stimulate narratives while allowing to maintain a fluid structure and interviewer flexibility.^1^ The interviewer did not provide definitions of what AI is or make further distinctions towards other forms of automated technologies, enabling interviewees to present their own understandings; only in the concluding questions were concrete use cases presented to the participants. All participants were familiar with AI technologies, with a majority reporting practical experiences with chatbots based on large language models in particular. While an increasing number of people are gaining experience with these technologies, the very high level of awareness could result from the aforementioned bias in the sample and a specific willingness to participate. It was also found that participants’ knowledges, expectations and concerns regarding algorithmic technologies were often informed by the controversial debate surrounding the introduction of a system for the automated segmentation of jobseekers.^2^ Although the interview guide explicitly refrained from referring to this debate to avoid possible biases, it was mentioned by participants in most interviews. Data analysis was based on inductive qualitative coding, the condensation of codes into categories and concepts, the writing of analytical and theoretical memos and rigid applications of iterative comparison inspired by grounded theory methodologies (Charmaz, 2014; Birks and Mills, 2023).

## 4 Analysis

Following the research question of how counselors in PES anticipate future AI technologies, a first step describes the ambivalent perceptions of ICTs proliferation, role transformations accompanied by restrictions of discretionary margins and experiences of dehumanization. Contrary to immediate expectations, it was found that these opinions resulted not in a rejection, but an affirmation of future technological trajectories through taming them. Discussing how present problems and speculative tensions are appropriated within tamed anticipated futures, a final step investigates the characteristics of such taming.

### 4.1 “Everything gets more and more complicated”

Counselors perceive ICTs in the context of PES ambivalently: alongside positive assessments centering opportunities to technologically improve work, prevailing opinions express reservations against existing systems, accounting them for the notion that “everything gets more and more complicated” (INT3). Technology is seen as reinforcing existing exclusions (e.g., based on age), creating dependencies, requiring the acquisition of knowledges and skills and enabling control through surveillance and datafied performance measures. Specifically, concerns were expressed that ICTs result in more rigorous regimes of work (increasing time pressure, more administrative work) that could lead to an excessive transfer of responsibility and decision-making power to digital systems. Despite one counselors’ conviction that “the computer is our most important professional tool” (INT23), ICTs are also demanding:

*“I have the feeling it’s getting more and more. […] There are always new functions where I then think to myself, how does this work again?” (INT16).*

These irritations result from the inconvenience associated with using in-house applications, where availability and reliability are prioritized at the expense of usability. Working with these applications is experienced as cumbersome and impractical as desired functions (such as searching or filtering data) are largely absent. Instead of comprehensive revisions, new political and organizational requirements to counseling processes are continuously implemented complementing existing legacy applications. Thus, imaginations of the system describe it as a “grape vine growing ever more grapes” (INT16), as “cancerous” (INT2), or, as a counselor in his twenties concluded: “there are some applications that are older than me” (INT14).

Despite these obstacles, digital systems are portrayed as inevitable for handling the increasing complexities of the labor market and the requirements arising from controlling, demanding for the statistical analyzability of key figures that aim at making placement services of the AMS “measurable,” both internally and politically. In this situation, ICTs manifest as cause and central driver of growing intricacies and simultaneously as necessary in order to face them.

### 4.2 Growing demands on “living form fields”

Counselors report feelings of increased pressured resulting from changing framework conditions: time constraints, scarcity of resources, but also a diversification of the job market, training opportunities and the resulting demands for specialized expertise and detailed domain knowledge. Allowing less focus on clients and tying up advisor’s attention, ICTs are identified as an obstruction, or, as one counselor stated: “I think that 80 percent of what I do is looking at the IT, looking at the CV, looking at reports, and 20 percent is talking to the customer. It really should not be like that” (INT6).

These tensions account for further-reaching transformations of professional roles, as expressed by an experienced counselor who had witnessed these upheavals during his career: “To be honest, yes, I am a living form field now, so to speak” (INT1). Once the core element of a successful counseling situation, caseworkers perceive their own agencies as threatened: as “operators” of systems that translate between machinic requirements and clients, the discretion of “street-level bureaucrats” is increasingly pushed back and changed towards responsibilities primarily concerned with mediating data between systems. Augmenting advisory activities with additional obligations to document cases and provide data for digital applications are problematized by caseworkers, reframing professional roles as performing “assembly line work” as part of a “fast food franchise” (INT18). A progressing standardization of work processes is reflected by a perceived “loss of humanity”:

*“At the moment, the human element is becoming less and less important because we constantly have to enter, document and review things. And I think that’s the wrong way to go. There really is too little actual counseling.” (INT13).*

This is accompanied by an erosion and devaluation of specialist domain knowledge: “one’s own brain,” as an experienced counselor remarked (INT20), is consumed by operating the systems, while “real counseling,” utilizing own knowledge and experience, continually decreases.

### 4.3 The computational loss of humanity

ICTs are perceived as failing to account for clients’ preferences and individual needs, not allowing for their “holistic” datafication and consideration of “human” aspects. Applications patronize (or correct) jobseekers (e.g., with regard to their education or language skills), require the completion of certain form fields and necessitate clear categorization, promoting the production of unambiguousness and eradicating former “grey areas.” Criticizing these inflexibilities with present matching systems, one counselor remarked:

*“I can look up professions that I can do, but what if I cannot do them for health reasons? Or what if I’m a shy person and it recommends something in retail or in a counseling setting?” (INT14).*

Rendering clients visible merely as collections of data points is seen as unable to consider people holistically and through their true “human” features. Attributing such dehumanizing inflexibility to automated systems is a crucial aspect within the appraisal of present technologies. Human skills of assessment, which are perceived to exceed any machinic and purely data-driven means of evaluation, are emphasized and positioned as being of central importance within present and future consulting situations. Counselors stress the need for rigor in the delivery of policies, while at the same time they highlight the decisive significance of discretionary margins that necessitate “human” consideration, discursively positioning themselves against the possibility of their own redundancy through automation:

*“We are strict. We have to adhere to our guidelines. But we are also human.” (INT2).*

Thus, notions of current technological infrastructures are characterized by perceptions of an ongoing proliferation of digital systems, a restriction of discretionary margins through the transformation of professional roles and a sense of data-driven dehumanization of the counseling situation. It seems plausible to assume that these views are then mirrored in rather pessimistic assessments of future technologies. However, and contrary to their outright rejection, the next section demonstrates how anticipations of AI futures are informed by current trends, but appropriate and thereby tamed.

### 4.4 AI as ambivalent empowerment

With counselors expecting an intensification of present trajectories, especially regarding the neglect of individual differences within automated decision-making that ignores specifically situated experiences, feelings, and ambitions of clients, critique on future AI systems is widespread. Despite these reservations, participants generally displayed considerable openness to technological changes and envision far-reaching potentials for AI use cases. Such included, for example, the review of legal guidelines, the automated evaluation of clients’ skills (e.g., based on their professional biography) and the issuing of recommendations for job, training, and education opportunities according to these assessments, the recording and compilation of clients’ data, the overcoming of barriers (e.g., languages, learning and reading difficulties) and performing administrative routine activities. Goals associated with the application of AI in these cases were saving time through simplification, allowing time for other tasks (especially talking to clients), achieving better results (tailored career advice, job and training placements) or enabling clients to more self-determination (by reducing dependency on opening hours and appointments).

Numerous configurations of roles and agencies result from these descriptions: future AI applications are perceived either as empowerment over (technological/institutional/legal) requirements or to overcome human limitations (knowledge about specific training opportunities or jobs). They are anticipated as a “helping hand,” as a “mediating instance” between stakeholders (clients, systems, guidelines), processes (matching and placing of jobseekers), people and their data, but centrally, between counselors and existing data/systems. Ironically, the systems currently perceived as too numerous and complicated are thus to be countered by expanding ICTs even further, with AI being aspired as empowerment over the technological status quo:

*“We are documenting more and more and often there is not enough time left over for customers. And that, I think, is the crux of the matter, especially in the advisory context of AI, that there really is a lot of potential to make our work easier with automation.” (INT23).*

Imaginations of AI contain boundaries regarding their capabilities: Crucially, the counseling situation is perceived as too individual, as influenced by too many parameters and framework conditions to simply become datafied and executed along automated procedures. Machines would be unable to understand the “essence” of counseling: “The facial expressions, the gestures, what the customer is saying to the other person, how should they be able to interpret that?” (INT13). Concerns expressed regarding the limited “human” capabilities of AI, coupled with an aspiration of future role configurations that emphasizes technological support over algorithmic paternalism resulted in the comprehensive agreement that final decisions over clients’ placements (in jobs or trainings) should not be left to automated systems. Human supervision was named as a central criterion for the acceptance of AI and key discursive strategy to argue against counselors’ future redundancy. Despite being formalized in many respects, counseling remains to have grey areas, ambiguities, and gaps that, in the manner of street-level bureaucrats, need to be “bridged.” Practicing what in the perception of caseworkers constitutes “good counseling” necessitates resorting to “human” skills that allow for clients to be regarded as a human being. It is feared that AI, strictly adhering to the rules without empathy, will further reduce margins of human assessment, erode counselors’ agencies and clients’ trust. This is also reflected in fears that customers will only be treated as “numbers” due to ongoing standardization and homogenization (of work processes, but also through predefined categories/form fields): “I always say that we should never be replaced, because the human element must always remain” (INT5).

At the same time, it is feared that expanding machinic agencies, growing time constraints, a loss of domain knowledge or an increasing pressure to achieve certain key figures may lead to counselors blindly following automated decisions without questioning them:

*“Artificial intelligence really will become more and more important, and people will think less and less about it. And at some point, our intelligence will probably diminish because we will not think about things at all or we’ll just rely on a system and take the convenient route.” (INT22)*

### 4.5 The taming of AI futures

However, and despite ambivalences, reservations and concerns over the anticipated consequences of AI’s future applications, counselors do not reject, but rather aspire to them. Envisioned to overcome today’s excessive efforts to deal with computational infrastructures, AI is anticipated to shift prevailing time regimes and simplify routine tasks. By automating data collection, documentation, and assisting in the pre-selection of options, the introduction of AI is believed to grant time for “real” and “human” counseling rather than operating systems. As liberated from present technological and institutional constraints, counselors aspire to re-establish the use of their “human” skills and affective expertise in the consulting situation. As “experts” evaluating and supervising automated suggestions through empathic accounts, counselors “tame” AI by projecting themselves into uncertain futures, articulate elements of practice that machines will be unable to adopt and reframe their professional identities according to this assessment.

Aspired future systems were described in the vocabulary of anthropomorphic assistant roles: While some wish for a “librarian” who prepares and curates information on request, the idea of a “helper” supporting counselors in the background (i.e., not directly interfacing clients) is particularly common. The metaphorical description as “kind of like a teacher. A corrector.” (INT15) evoked more authoritative associations, however, was relativized later to again serve the narrative of background support: “It would just be kind of practical. If someone says, hey, that does not fit, or hey, you have to do it differently.” (INT15).

As anticipations oscillate between machinic possibilities and the limits of automation, taming addresses and mitigates potentially disruptive effects: counselors order insecurities and concerns they expect to manifest through AI technologies not by rejecting, dismissing, or neglecting seemingly inevitable developments, but by appropriating them. Since the alternative, namely accepting “untamed” AI and its discursively produced superiority threatening “humaneness,” inevitably results in counselors’ redundancy, a mutual approximation of humans and machines preserves their necessity. Taming then results in an empowerment over current constraints and overcomes perceived tensions and anticipated uncertainties; it does not deny transformations, but functions as a solution to encounter expected disruptions and their unforeseeable effects.

Participants expected uncertain futures arising from and relating to currently perceived problems. However, instead of deriving their own redundancy from this or adopting a defiant position, trajectories of potentially disruptive technologies were instead adjusted, defused and ultimately tamed. Such anticipations were both diverse and contradictory, depicting future AI simultaneously as empowerment and authority, as support and restriction or as overcoming human limits while imposing novel barriers. However, despite their wide range of imaginations, tamed futures contain corresponding elements that were identified to distinguish taming from other forms of anticipation: dependencies, dialectics, and demarcations.

#### 4.5.1 Dependencies

Already Saint-Exupéry’s fox knew about the mutual dependence that taming entails: “if you tame me, then we shall need each other” (1971, p. 80). And indeed, the taming of anticipated AI futures intrinsically relies on expanding human-machine relationships, albeit under different circumstances:

*“I would like to talk to the customer in a meaningful way and not have to look at the computer all the time. […] I would really like to be able to talk to the customer more and get a decision [based on prior inputs] and then explain it to the customer. And that’s a huge opportunity for me with AI.” (INT6).*

Rather than dismissing AI, taming builds on subjecting its future possibilities and proposes them as remedies to a currently perceived situation of entrapment. Tamed futures depend on both enhanced technological capabilities and the humanity of their operators, linking discursively shaped expectations around technologies and aspirations of future working conditions.

#### 4.5.2 Dialectics

What is described here as taming of futures not simply extrapolates present tensions. It transcends them by overcoming their contradictions. Inherent to this way of thinking is that a perceived “dehumanization” occurs not in isolation, but as an initial proposition and in relation to what are regarded desirable, humane working conditions. A distinctive aspect of taming is that these conflicting opposites do not result in the acceptance of seemingly predictable trajectories or a resignation before the circumstances. They relate to each other as thesis and antithesis within the triadic structure typical for dialectic thinking and dissolve into a synthesis: a future that is tamed. Emerging from this conflicting relation, tamed futures act as “speculative” or “positively rational” (Hegel, 1830, paras. 79, 82) that integrate ostensible antagonists. Or, in the terminology of Hegelian (1830) dialectic, as mutual sublation (*Aufhebung*): Insisting on the need for “humaneness” within a “dehumanized” counseling situation, the *abstract* (confined present working conditions) is mediated through its *negative* (seemingly inhuman AI) and results in a *concrete*: futures that overcome contradictions while at the same time preserving and maintaining their initial conditions. The “synthesized” versions of these futures consist of both their (human) thesis and their (technological) antithesis, but moves both beyond their inherent limitations: by achieving more humaneness through machinic means, gaining autonomy through a higher dependence on digital infrastructures, and simplifying the complexities of institutional demands through an additional layer of complexity.

Tamed AI futures sublate the status quo in which they are rooted by promoting the very technologies that are considered the decisive reason for current intricacies. Taming does therefore not fundamentally deny transformations, but functions as a solution to encounter both expected disruptions and their unforeseeable effects. At the same time, it answers to and transcends the perceived flaws of emerging system-level bureaucracies. To tame futures dialectically with and by anticipating certain algorithmic technologies means affirming ideas of supposedly better working conditions (more humanity and margin of discretion) and regaining (or even improving) them by the means that are held responsible for their loss. Negating an initial contradiction within a “unity of terms (propositions) in their opposition” (Hegel, 1830, para. 82), taming is not an “empty and abstract nothing” (Hegel, 1830, para. 82), but affirms the disintegration and transition of antagonists.

#### 4.5.3 Demarcations

Appropriations of futures can be considered in terms of the boundaries that define which aspects of anticipated situations (technologies, institutions, regimes) are considered “tamable” (and which are not). Applying this lens revealed how AI futures respond to perceived issues of techno-bureaucratic regimes, but in their consequences aim at alleviating rather than overcoming prevailing conditions. In the particular case explored, institutional structures that were perceived as both criticizable and inevitable led to anticipations that applied a symptomatic treatment of problems through technological innovation without systematically resolving or subverting them. Positioning AI as empowerment over current entrapments, taming appropriates and thus defuses certain problematic trajectories, however, without transcending the logics of bureaucratic regimes responsible for them comprehensively.

## 5 Conclusion

Experienced disempowerment, loss of formerly perceived agency, demands resulting from an increased use of technological systems and accompanying notions of dehumanization are decisive for anticipating the effects of future AI applications. Such futures are not abstract, continuous, exchangeable and to be “traded” for another, but embodied, embedded and contextual, attached to unique being and events (Adam and Groves, 2007, p. 204); they form and inform links between current transformations and expected future developments.

Taming has been offered as an analytical lens to perceive the manifold practices of workers’ anticipation that occur within the institutional limits of bureaucratic organizations and as “cultural practices in response to the problem of transience, uncertainty and indeterminacy” (Adam and Groves, 2007, p. 39). As an attempt to regain discretionary margins eroded by digital transformations, taming has been described as a tactic to appropriate uncertain futures against understandings and discourses that would presume defensive stances or even neo-luddist tendencies within a workforce facing threats of being automatized by AI. Taming emphasizes contingent negotiations of preferable and desirable futures by anticipating empowerment over a perceived status quo. Understanding such anticipating “as a form of resistance against hegemonic forces” (Markham, 2021, p. 8) should, however, not reproduce binary assumptions of “compliance” and “resistance.” Rather, the perspective highlights the complexities that are involved within these processes: As inherently dialectic, taming serves as both synthesizing seemingly contradictory elements within speculative futures, establishes mutual dependencies, and demarcates aspects that appear as tamable from those that are not. It is precisely “the affirmative, which is involved in their disintegration and in their transition” (Hegel, 1830, para. 82) that sets taming apart from similar adaptive strategies: it is a way of coping that, instead of resigning to an apparent threat of automation, envisions futures in which menaces are acknowledged, yet at the same time are “domesticated” (Silverstone, 1994). But unlike uniliteral embedding of technology into existing domestic environments, such contestation also requires those that resist to adapt: it “means to establish ties” (Saint-Exupéry, 1971, p. 80). While taming resists algorithmic transformations, it is also “productive” (Ettlinger, 2018). It makes use of and subverts existing power relations and their materialized institutions by “constructing” and “building new social facts and relations” (Baaz et al., 2023, p. 72). Rather than “avoiding” algorithmic power or “breaking” the threat of automation, taming constructs futures that chart ways out of a perceived technological entrapment.

Despite, or perhaps precisely because of the synthesis of contradictions, taming does not produce “counter-imaginaries” that “animate civil society’s tactical responses to perceived threats to its values and ways of living” (Kazansky and Milan, 2021, p. 366). It does not mobilize against expected problems, does not call for action against future threats, or produces (counter) publics (Marres, 2015). Rather, it is a way of coping within an institutional framework without overcoming or subverting it. Tamed futures serve as self-affirmations of counselors’ future necessity within a realm threatened by technologies, while, at the same time, they affirm and intensify technological dependencies. Rather than trying to “bulwark autonomy, increase agency, and provoke critical inquiry into new ways of being and doing” (Kazansky and Milan, 2021, p. 376), taming futures does not oppose dominant imaginaries of datafication, but tackles present contradictions dialectically: by aspiring for gaining autonomy through dependence on digital infrastructures, humanity through machinic means, and simplification through additional complexity. Resulting from and building on common dichotomies of humans vs. machines, compliance vs. resistance or autonomy vs. dependence, taming transforms them toward their mutual sublation.

However, what is described here as the taming of uncertain futures emerged within the PES agency of the Austrian digital welfare state and thus along particular institutional, legal, organizational and bureaucratic frameworks and spatio-temporal configurations, in which knowledges about the potential impacts of AI were available, but practical experiences largely absent. Further limitations result from the singular time of the data collection and a resulting neglect of longitudinal observations which, in addition to a comparison across national systems, should be taken into account in future empirical studies. While taming conceptualizes a specific analytical perspective on the negotiation of AI futures, central questions, e.g., regarding its applicability within other institutional contexts, the conditions and reasons that determine how anticipations are tamed or effects on technologies’ actual future “domestication” (Silverstone, 1994), remain unclear. Additionally, counselors’ efforts of taming must be further considered in relation to factors that were identified as obstacles (e.g., “algorithmic aversion,” Dietvorst et al., 2015; “institutional inertia,” Aksom, 2022) or affirmations (e.g., “automation bias,” Alon-Barkat and Busuioc, 2023) to the algorithmic transformation of practices and organizations. While taming was introduced as a concept to analyze the content and structure of sociodigital futures, further research is needed to clarify the specific actions and routines that emerge from it: how taming remakes the present and sabotages the future.

Taming seeks to sensitize towards analyzing the in- and exclusions that it encompasses, i.e., what aspects of a situation are being “tamed” or perceived to be “tamable”: counselors’ aspirational scenarios mainly involved “enacting the state” through affective labor (Penz et al., 2017) aided by technologies without questioning the prevailing regimes of activation that require and demand such labor from counselors. Therefore, workers’ tamed anticipations envision AI futures and the human-machine configurations to come “from below” (rather than merely reproducing hegemonic state- or company-driven imaginaries, Jasanoff and Kim, 2009; Mager and Katzenbach, 2021), while perpetuating discursive closures that frame their modalities of imagination (Markham, 2021).

## Data Availability

Despite careful anonymization, participants’ informed consent was only obtained for using data in the context of the study. No further use or release is possible.

## Footnotes

^1^Such included for example: “Please tell me how a typical consulting situation works. How and for which tasks do you use certain technologies (e.g., applications or databases)” or “How do you think new technologies will change your work as a consultant in the next years?.”

^2^Promoted as supporting counselors to evaluate and classify clients’ support requirements, the system was stopped after a trial period in 2020. Problematizing the “neutrality” and “objectivity” of the planed system sparked academic debates and initiated broad public negotiations of algorithmic technologies in Austria (Allhutter et al., 2020; Berner and Schüll, 2020). Discussions were driven by concerns around algorithmic fairness in public services and a resulting dehumanization of state welfare institutions (Eubanks, 2017; Desiere and Struyven, 2021; Mitchell et al., 2021).
